# Unraveling the kinetics and molecular mechanism of gas phase pyrolysis of cubane to [8]annulene

**DOI:** 10.1039/d0ra05371f

**Published:** 2020-09-04

**Authors:** Ahmad Seif, Luis R. Domingo, Ehsan Zahedi, Temer S. Ahmadi, Elham Mazarei

**Affiliations:** Department of Chemistry, Central Tehran Branch, Islamic Azad University Tehran Iran Ahmaseif@yahoo.com; Department of Organic Chemistry, University of Valencia Dr. Moliner 50, 46100 Burjassot Valencia Spain; Department of Chemistry, Shahrood Branch, Islamic Azad University Shahrood Iran; Department of Chemistry, Villanova University Villanova PA 19085 USA

## Abstract

The kinetic and electron density flows are studied theoretically for the gas phase pyrolysis of cubane *via* its cage opening to reach bicyclooctatriene and then thermal rearrangement of bicyclooctatriene to produce [8]annulene which is the experimentally observed major product. The observed kinetic data at the MN15-L/maug-cc-pVTZ level of theory were in good agreement with the experimental results as compared to the CBS-QB3 method. The cage opening and the thermal rearrangement steps at the experimentally employed temperature of 520 K were exergonic and exothermic. The atmospheric rate constants calculated by means of the RRKM theory show that the cage opening is the rate-determining step. The temperature dependence of the rate constant for the cage opening step at the MN15-L level can be expressed as log(*k*/s^−1^)^1bar^_MN15-L_ = (15.63) − (48.99 kcal mol^−1^)/*RT* ln 10. The molecular mechanism of the reactions has been investigated by means of the bonding evolution theory (BET) at the B3LYP/6-311G (d,p) level of theory. The cage opening course is described topologically by cleaving of C1–C2, C4–C8, and C5–C6 single bonds and electron saturation of the C1–C4, C2–C6, and C5–C8 bonds, while the rearrangement of bicyclooctatriene is described by C3–C7 bond rupture, depopulation of C1–C4 and C5–C8 double bonds, and electron saturation of C1–C5, C3–C4, and C7–C8 bonds. Electron density rearrangement along the two successive steps are asynchronous and the sequence of catastrophes can be represented as: *η*-1-13-C^†^C^†^FFFC^†^C^†^FFFC^†^C^†^-2-6-[C]_2_C^†^[F]_2_[C^†^]_2_C^†^-0.

## Abstract 2

Kinetic and electron density flows are studied theoretically for the gas phase pyrolysis of cubane via its cage opening to bicyclooctatriene and then thermal rearrangement of bicyclooctatriene to produce [8]annulene which is the experimentally observed major product.

## Graphical abstract

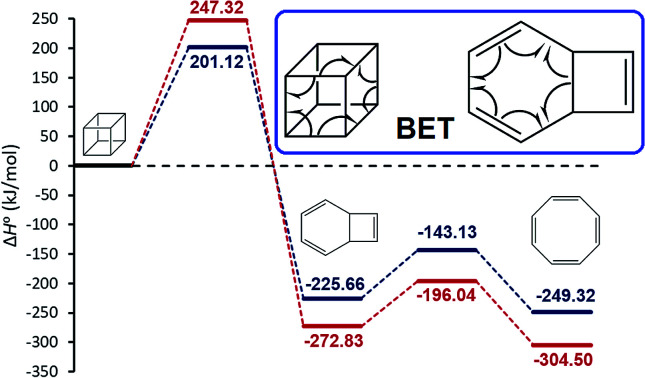

## Introduction

The fascination of the structure and unusual physicochemical properties of the non-natural compounds have motivated scientists to focus on impossible compounds – those that break the classical rules of chemistry. Cubane is a landmark in the world of impossible compounds because of its exceptional structure, strain, and symmetry.^1^ The C–C–C bond angle of 90° in cubane is distinct from the usual value of ∼109.5° for sp^3^-hybridized carbon atoms and prevents rotation about the single bonds.^2^ Its exceptional structure makes cubane an immensely strained cage-like molecule with octahedral point group (*O*_h_) and cubic symmetry.^3^ Therefore, before the first successful synthesis of cubane in 1964,^4^ it was hard to believe that such a molecule could exist.^5^ Despite the strained cage, cubane is kinetically stable up to 220 °C.^6,7^ Martin *et al.*^8^ experimentally verified that no decomposition occurs below 200 °C and releasing of the strain during the decomposition process leads to the generation of highly vibrationally excited products; their work also revealed that the [8]annulene is the major product of pyrolysis at low pressures and the obtained Arrhenius equation for the pyrolysis of cubane in the temperature range 230–260 °C was log(*k*/s^−1^) = (14.68 ± 0.44) − (43.1 ± 1.0 kcal mol^−1^)/*RT* ln 10.^8^ They also pledged to report decomposition rate using the Rice–Ramsperger–Kassel–Marcus (RRKM) calculations but was not done so.

Zhang *et al.*^9^ have studied the infrared vibrational spectra, thermodynamic properties, and pyrolysis mechanism of octanitrocubane. They proposed that the pyrolysis mechanism of cubane takes place *via* two consecutive C–C bond homolysis to produce *syn*-tricyclooctadiene. They found that the activation energies for the first and second bond breaking are 117.71 and 20.68 kJ mol^−1^ calculated at the MINDO/3 level of theory, respectively.

Decomposition products of cubane and methylcubane were studied by Li *et al.*^10^ in a micro-flow tube reactor using collision induced dissociation mass spectrometry from room temperature to 1000 K. They identified a set of pyrolysis products and found that the effect of methyl functional group on the stability of cubane is insignificant. They also studied the electronic structure and energetic stability of species using the quantum chemical calculations. Maslov *et al.*^11^ studied the solid phase thermal stability of cubane at 1050–2000 K using Molecular dynamics simulations. They investigated the atom displacement during the decomposition of cubane to [8]annulene, benzene, and acetylene. They showed that the calculated solid phase decomposition activation energy 1.9 ± 0.1 eV (43.81 ± 2.30) kcal mol^−1^ is in good agreement with the reported experimental value.^8^ In a similar work, the influence of methyl functional group on the stability of solid cubane has been studied by Katin *et. al.*^12^ using molecular dynamics simulation at 900–1700 K, who showed that the solid phase activation energy of octamethylcubane (1.45 ± 0.21 eV) is smaller than that for cubane. They also indicated that methyl group can destabilize the solid state cubane but less than nitro group.

Recently, Shyamala *et al.*^2^ studied various decomposition pathways of cubane by means of quantum chemical calculations; they proposed various decomposition pathways and subsequent reactions based on the previous experimental findings. They reported heat of formation for all species; activation enthalpies and activation Gibbs free energies for all studied channels. They also analyzed the mole fraction of various species at different reactor wall temperatures. Even though the authors have pointed out quantum tunneling and rate constant of reactions, these parameters were not reported. Although the pyrolysis of cubane has been investigated experimentally and theoretically, the pressure- and temperature-dependence of the rate constants and bond formation/breaking of cubane along the decomposition pathways have not been investigated.

**Scheme 1:**
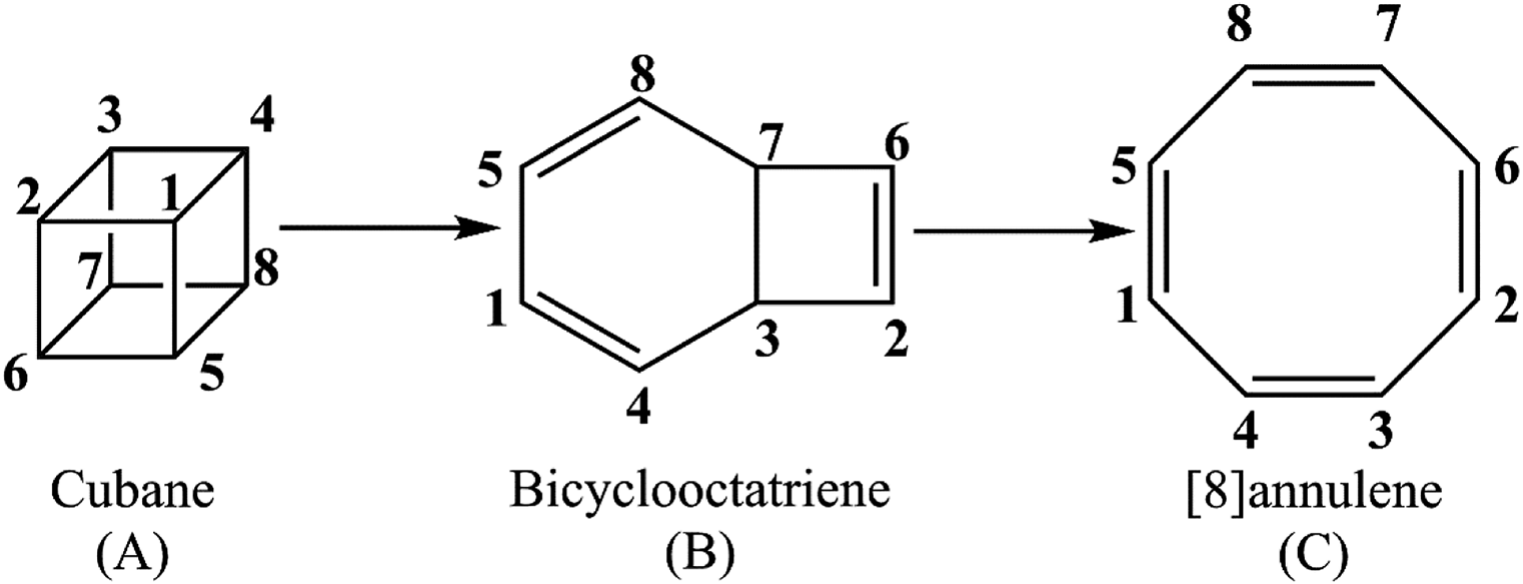
Details of calculation.

The present study offers a detailed kinetic study into the pressure- and temperature-dependence of the rate constants for the gas phase pyrolysis of cubane by means of the RRKM calculations and identifying the elementary chemical processes along the pyrolysis of cubane from the perspective of bonding evolution theory (BET).^13^ The later allows us to address how and where bonds are formed or cleaved as well as the electron pair rearrangements. Since [8]annulene is the major product of the cubane pyrolysis,^2,8^ we have constrained our study to the pathway which includes the formation of [8]annulene *via* the bicyclooctatriene intermediate (see Scheme 1).

The hybrid density functional B3LYP level with a standard 6-311G(d,p) basis set has been used for structural optimization of all stationary points and intrinsic reaction coordinate (IRC) calculations with step size 0.02 amu^1/2^ Bohr in mass-weighted Cartesian coordinates. The validity of the transition structures has been verified by frequency calculations at the same level of theory and the IRC method. The optimized transition structures had an imaginary frequency corresponding to the reaction coordinate and the structures were connected to the correct minima. The composite CBS-QB3 (ref. 14 and 15) method and local exchange-correlation functional MN15-L^16^ in combination with the minimally aug-cc-pVTZ^17^ basis set have been used to obtain highly accurate kinetic results. The MN15-L is a new local exchange-correlation functional and its accuracy is in good agreement with the high-level methods. The computed vibrational frequencies at the MN15-L/maug-cc-pVTZ level of theory were scaled by a factor of 0.979.^18^ The computational procedure in the CBS-QB3 method has been described elsewhere.^19,20^ All quantum chemical calculations were carried out with the Gaussian 16 suite of programs.^21^ The pressure- and temperature-dependent rate constants as well as thermodynamical parameters have been evaluated using the Kinetic and Statistical Thermodynamical Package (KiSThelP, Rev. 2019).^22^ The Lennard-Jones parameters for cubane, *σ* = 5.5 Å, *ε*/*k*_B_ = 442.0 K, and bicyclooctatriene, *σ* = 5.6 Å, *ε*/*k*_B_ = 491.3 K, were estimated from the relation given by Chung *et al.*^23^ and critical properties from the Joback–Reid method.^24^ Lennard-Jones potential parameters for nitrogen as buffer gas were *σ* = 3.738 Å, *ε*/*k*_B_ = 82.0 K.^25^ The collisional efficiency per unit collision *β*_c_ was assumed to be 0.15 for weak collisions (*β*_c_ < 1).^26^ For the BET analysis, the electron localization function's (ELF)^27^ calculations were performed for all the stationary points and each point on the IRC profile by means of TopMod package^28^ with a cubical grid of step size 0.04 Bohr on the B3LYP/6-311G(d,p) monodeterminantal wavefunctions. The ELF localization domains for all the stationary points and turning points connecting the successive structural stability domains (SSD) were visualized using the UCFC Chimera program.^29^

## Results and discussion

**Fig. 1:**
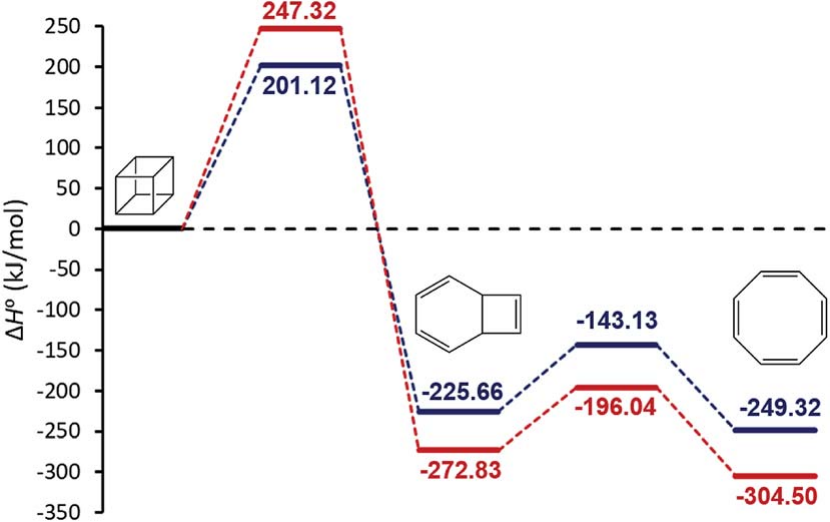
The relative gas-phase enthalpies of the stationary points on the PES for the formation of [8]annulene from the gas phase decomposition of cubane calculated at the MN15-L/maug-cc-pVTZ (blue) and CBS-QB3 (red) level of theories.

We begin our discussion by investigation of the thermodynamic and kinetic parameters to explore the energetic nature and rate-determining step for the formation of [8]annulene from the gas phase decomposition of cubane. The stationary points on the potential energy surface (PES) for the gas phase pyrolysis of cubane to [8]annulene are shown in Fig. 1. Table 1 summarizes thermodynamic parameters at the employed experimental temperature 520 K. The cage opening of cubane (A → B) is exergonic ranging from −259 to −305 kJ mol^−1^ and exothermic ranging from −225 to −272 kJ mol^−1^ depending on the employed computational method. High positive value of the entropy change for the cage opening step is due to the cage strain release. Therefore, the cage opening step is an enthalpy and entropy driven process. The thermal rearrangement of bicyclooctatriene (B → C) is an enthalpy driven process but involves a low entropy of the process. This step is also exergonic and exothermic ranging from −23 to −31 kJ mol^−1^, depending on the employed computational method.

**Table 1:** Thermodynamic parameters of the reaction including equilibrium constant, *K*, Gibbs free energy, Δ*G*^0^, enthalpy, Δ*H*^0^, and entropy, Δ*S*^0^, for the gas phase pyrolysis of cubane to [8]annulene calculated at 1 bar and 520 K^a^

Method	Reaction	*K*	Δ*G*^0^ (kJ mol^−1^)	Δ*H*^0^ (kJ mol^−1^)	Δ*S*^0^ (J mol^−1^ K^−1^)
MN15-L	A → B	1.06 × 10^26^	−259.09	−225.66	64.29
CBS-QB3		4.20 × 10^30^	−304.87	−272.83	61.66
MN15-L	B → C	230	−23.52	−23.66	−0.28
CBS-QB3		1520	−31.67	−31.67	−0.24

a For the ease of notations, cubane, bicyclooctatriene, and [8]annulene are denoted as A, B, and C, respectively.

**Fig. 2:**
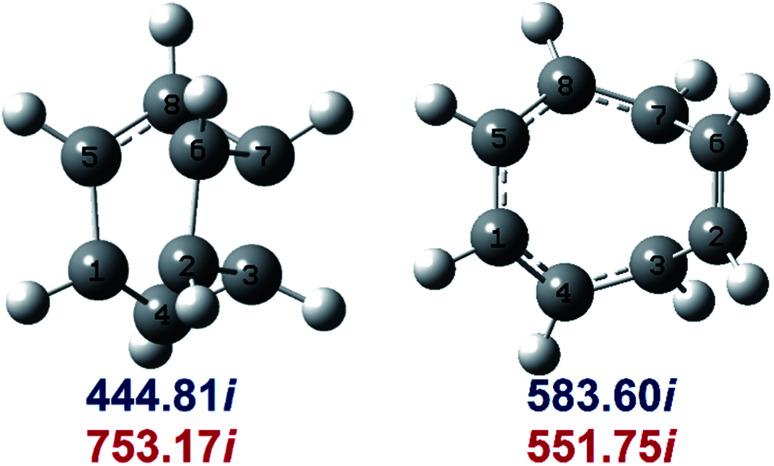
MN15-L/maug-cc-pVTZ geometries of TS1 (left) and TS2 (right). Imaginary frequencies of saddle points are computed at the MN15-L/maug-cc-pVTZ (blue) and B3LYP/CBSB7 (red), level of theories.

**Fig. 3:**
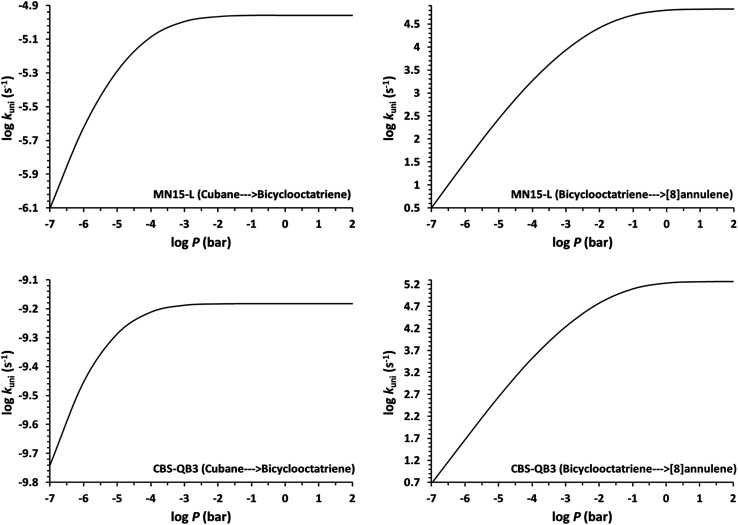
Fall-off curves as a function of pressure for the studied reactions calculated at 520 K.

Determination of the rate constants have been performed for atmospheric pressure with nitrogen as the buffer gas. The MN15-L/maug-cc-pVTZ geometrical structure for the saddle points associated with the cage opening step (TS1) and thermal rearrangement step (TS2) as well as imaginary frequency values are presented in Fig. 2. Fig. 3 illustrates the calculated pressure dependence of the rate constants at 520 K and the details of the full-off curves are tabulated in Table 2.

**Table 2:** Wigner tunneling correction (*κ*), high pressure limit of the rate constant (*k*_∞_), unimolecular rate constant at 1 bar (*k*^1bar^), low pressure limit of the rate constant (*k*_o_), and Lennard-Jones collision frequency (*Z*_LJ_) for the gas phase pyrolysis of cubane to [8]annulene calculated at 520 K^a^

Method	Reaction	*κ*	*k*_∞_ (s^−1^)	*k* ^1bar^ (s^−1^)	*k*_o_ (cm^3^ per molecule per s)	*Z*_LJ_ (cm^3^ per molecule per s)
MN15-L	A → B	1.06	1.09 × 10^−5^	1.09 × 10^−5^	1.78 × 10^−18^	5.36 × 10^−10^
CBS-QB3		1.18	6.56 × 10^−10^	6.56 × 10^−10^	2.94 × 10^−21^	
MN15-L	B → C	1.10	6.70 × 10^4^	6.31 × 10^4^	2.26 × 10^−12^	5.56 × 10^−10^
CBS-QB3		1.10	1.84 × 10^5^	1.69 × 10^5^	3.34 × 10^−12^	

a For the ease of notations, cubane, bicyclooctatriene, and [8]annulene are denoted as A, B, and C, respectively.

The results show that for the cage opening step, there is almost no atmospheric pressure dependence. As a result of the inefficiency of the transition state theory, the atmospheric pressure of the thermal rearrangement rate constant was found in the fall-off region, therefore use of the RRKM formalism to calculate the atmospheric rate constant is inevitable. Since *k*_B⃑C_≫*k*_A⃑B_, the concentration of the bicyclooctatriene is low and essentially constant, implying that the cage opening is the rate-determining step. The temperature *dependence* of atmospheric *rate constants* over the temperature range 500–530 K are calculated by the linear fitting of the rate constants against inverse temperature to the Arrhenius equation. The fitted Arrhenius parameters as well as thermochemical activation data are presented in Table 3.

**Table 3:** The fitted Arrhenius parameters (A: pre-exponential factor; *E*_a_: activation energy) for the calculated unimolecular rate constant at 1 bar over the temperature range 500–530 K at 5 K intervals. Activation Gibbs free energy, Δ*G*^‡^, activation enthalpy, Δ*H*^‡^, and activation entropy, Δ*S*^‡^, are calculated at 520 K^a^

Method	Reaction	Log *A* (s^−1^)	*E*_a_ (kcal mol^−1^)	Δ*G*^‡^ (kJ mol^−1^)	Δ*H*^‡^ (kJ mol^−1^)	Δ*S*^‡^ (J mol^−1^ K^−1^)
MN15-L	A → B	15.63	48.99	193.28	201.12	15.07
CBS-QB3		16.09	60.14	235.77	247.32	22.22
MN15-L	B → C	13.32	20.30	82.26	82.53	0.51
CBS-QB3		13.13	18.82	77.91	76.79	−2.14

a For the ease of notations, cubane, bicyclooctatriene, and [8]annulene are denoted as A, B, and C, respectively.

It is evident that the rate-determining step *controls* the *rate* of the entire process. A linear least-squares fit to the cage opening step values correspond to the Arrhenius equations:log(*k*/s^−1^)^1bar^_MN15-L_ = (15.63) − (48.99 kcal mol^−1^)/*RT* ln 10andlog(*k*/s^−1^)^1bar^_CBS-QB3_=(16.09) − (60.14 kcal mol^−1^)/*RT* ln 10

The obtained Arrhenius equation from the MN15-L/maug-cc-pVTZ level is closer to the experimental results^8,11^ as compared to the CBS-QB3 calculated values.

**Fig. 4:**
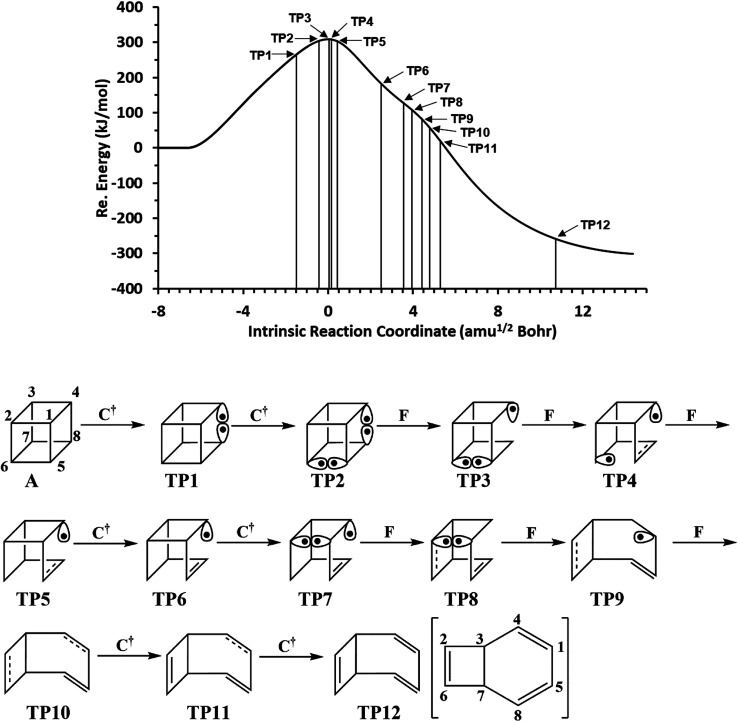
The IRC profile for the cage opening step calculated at the B3LYP/6-311G(d,p) level of theory with marked turning points and classical representation of the reaction mechanism.

**Fig. 5:**
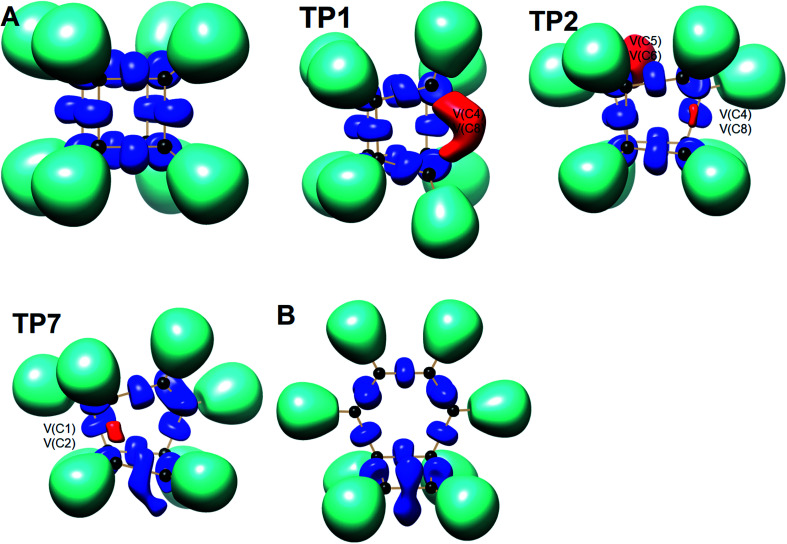
Snapshot of ELF-localization domains (*η* = 0.80) for cubane, bicyclooctatriene, and turning points associated with the bond breaking in the cage opening step.

We have also explored the sequence of chemical events along the cage opening and thermal rearrangement reaction pathways. The BET study along the cage opening pathway shows that the reaction process takes place along the 13 SSDs. The topological division of the energy profile and the sequential bonding changes along the IRC are presented in Fig. 4. The snapshot of ELF localization domains for cubane, bicyclooctatriene, and turning points associated with the bond breaking are depicted in Fig. 5. The cubane molecule can be described topologically by twelve single disynaptic basins. Due to the cubane symmetry, the populations for all (Ci,Cj) disynaptic basins were 1.85 ē, equal to the ELF-topological bond order 0.92, which is slightly smaller than the formal value of 2 ē. The basin populations are presented in Table 4. Two consecutive turning points, TP1 and TP2, along the reaction path appear at *s* ≈ −1.48617 and *s* ≈ −0.43146 amu^1/2^ Bohr by means of two consecutive cusp (C^†^C^†^) type catastrophes. Each one of two disynaptic basins V(C4,C8) and V(C5,C6) are split into two monosynaptic basins located on the respective carbon atoms. The first SSD is the most energetic with an energetic cost of 266.36 kJ mol^−1^ associated with the formation of V(C4) and V(C8) monosynaptic basins which their populations at TP1 are 1.15 and 0.58 ē, respectively. The basin populations for the V(C5) and V(C6) monosynaptic basins at TP2 are 0.58 and 0.49 ē, respectively. From a chemical point of view, the mentioned changes are associated with the rupture of the C4–C8 and C5–C6 single bonds, respectively, as well as formation of pseudoradical centers at the interacting carbons. Subsequently, three consecutive turning points TP3-TP5 at *s* ≈ 0.04795, *s* ≈ 0.14382, and *s* ≈ 0.43146 amu^1/2^ Bohr, respectively, by means of fold (FFF) type catastrophes yield annihilation of the V(C8), V(C5), and V(C6) monosynaptic basins, respectively. This indicates that the pseudoradical centers at the C8, C5, and C6 atoms are depopulated due to the growing disynaptic basins and consequently disappear. Along the SSD III, the V(C8) monosynaptic basin is completely depopulated for the V(C5,C8) disynaptic basin with basin population 2.91 ē at TP3. Similarly, SSD IV associated with the complete depopulation of V(C5) monosynaptic basin to the V(C5,C8) disynaptic basin with basin population 3.23 ē at TP4. In the next domain (SSD V), the formed V(C6) monosynaptic basin at TP2 is completely depopulated to the V(C2,C6) disynaptic basin with basin population 2.46 ē at TP5. The TP6 connecting SSD VI and SSD VII takes place at *s* ≈ 2.49294 amu^1/2^ Bohr by means of a cusp (C^†^) type catastrophe. The topological signature of the single C5–C8 bond has been changed to the double C5=C8 bond by transformation of single disynaptic basin V(C5,C8) into the pairs of V_1,2_(C5,C8) integrating to a total of 3.32 ē. Meanwhile, the V(C4) monosynaptic basin is depopulated to the V(C2,C6) disynaptic basin with basin population 2.72 ē at the beginning of this domain. The next turning point, TP7, occurs between SSD VII and SSD VIII at *s* ≈ 3.54764 amu^1/2^ Bohr. This turning point is predicted to be a cusp (C^†^) type catastrophe as a transformation of disynaptic basin V(C1,C2) into two monosynaptic basins V(C1) and V(C2) with basin populations 0.61 ē and 0.59 ē, respectively, which are localized on the respective atoms. Chemically, the respective turning point is associated with to the breaking of C1–C2 single bond and formation of pseudoradical centers at the C1 and C2 atoms. An important event in this domain is electron density transfer from the V(C4) monosynaptic basin to the V(C1,C4) and V(C2,C6) disynaptic basins with basin populations 2.37 ē and 2.94 ē, respectively, at the TP7. Repeatedly, three successive turning points TP8-TP10 take place at *s* ≈ 3.93116, *s* ≈ 4.41057, and *s* ≈ 4.79410 amu^1/2^ Bohr, respectively. The relevant turning points indicate threefold successive (FFF) type catastrophes associated with the annihilation of monosynaptic basins V(C4), V(C2), and V(C1), respectively, which are associated with the disappearing of pseudoradical centers at the C4, C2, and C1 atoms. Along the SSDs VIII, IX, and X, the monosynaptic basins V(C4), V(C2), and V(C1) are completely depopulated to the disynaptic basins V(C1,C4), V(C2,C6), V(C1,C4), respectively, with the basin populations 2.75 ē, 3.35 ē, and 3.12 ē at the relevant turning points. Finally, two successive turning points occur at *s* ≈ 5.27351 and *s* ≈ 10.73887 amu^1/2^ Bohr by means of two consecutive cusp (C^†^C^†^) type catastrophes.

**Table 4:** Population of the ELF-localization basins associated with the cage opening step calculated at the B3LYP/6-311G(d,p) level

	A	TP_1_	TP_2_	TP_3_	TP_4_	TP_5_	TP_6_	TP7	TP8	TP9	TP10	TP11	TP12	B
V(C_1_,C_2_)	1.85	1.76	1.71	1.68	1.67	1.64	1.42	—	—	—	—	—	—	—
V_1_(C_1_,C_4_)	1.85	1.88	1.87	1.87	1.88	1.90	2.16	2.37	2.75	2.80	3.12	3.08	1.74	1.75
V_2_(C_1_,C_4_)	—	—	—	—	—	—	—	—	—	—	—	—	1.57	1.65
V(C_1_,C_5_)	1.85	1.95	1.98	1.99	2.00	2.00	2.02	2.03	2.04	2.09	2.15	2.21	2.29	2.22
V(C_2_,C_3_)	1.85	1.80	1.81	1.82	1.82	1.83	1.81	1.82	1.83	1.85	1.86	1.88	1.92	1.95
V_1_(C_2_,C_6_)	1.85	1.96	2.04	2.10	2.11	2.46	2.72	2.94	3.03	3.35	3.42	1.89	1.73	1.74
V_2_(C_2_,C_6_)	—	—	—	—	—	—	—	—	—	—	—	1.63	1.71	1.70
V(C_3_,C_4_)	1.85	1.90	1.89	1.89	1.89	1.90	2.00	2.09	2.12	2.14	2.13	2.13	2.07	2.04
V(C_3_,C_7_)	1.85	1.88	1.87	1.86	1.85	1.84	1.79	1.77	1.76	1.75	1.74	1.73	1.75	1.80
V(C_4_,C_8_)	1.85	—	—	—	—	—	—	—	—	—	—	—	—	—
V(C_5_,C_6_)	1.85	1.46	—	—	—	—	—	—	—	—	—	—	—	—
V_1_(C_5_,C_8_)	1.85	2.07	2.29	2.91	3.23	3.27	1.72	1.76	1.76	1.77	1.77	1.76	1.75	1.75
V_2_(C_5_,C_8_)	—	—	—	—	—	—	1.60	1.66	1.69	1.71	1.72	1.71	1.57	1.65
V(C_6_,C_7_)	1.85	1.83	1.89	1.93	1.93	1.96	2.01	1.96	1.96	1.95	1.94	1.93	1.93	1.95
V(C_7_,C_8_)	1.85	1.94	1.96	1.97	1.98	1.99	1.99	1.99	1.99	2.00	2.01	2.02	2.07	2.04
V(C_1_)	—	—	—	—	—	—	—	0.61	0.52	0.39	—	—	—	—
V(C_2_)	—	—	—	—	—	—	—	0.59	0.52	—	—	—	—	—
V(C_4_)	—	1.15	1.24	1.29	1.30	1.30	0.77	0.39	—	—	—	—	—	—
V(C_5_)	—	—	0.58	0.38	—	—	—	—	—	—	—	—	—	—
V(C_6_)	—	—	0.49	0.38	0.34	—	—	—	—	—	—	—	—	—
V(C_8_)	—	0.58	0.53	—	—	—	—	—	—	—	—	—	—	—

The related turning points are characterized by splitting of V(C2,C6) and V(C1,C4) disynaptic basins into the pairs of V_1,2_(C2,C6) integrating to a total of 3.52 ē and the pairs of V_1,2_(C1,C4) integrating to a total of 3.31 ē-associated with the change in the topological signature of the C2–C6 and C1–C4 single bonds to double bonds. Along domains XII and XIII, relaxation of the electronic structure occurs until the bicyclooctatriene electronic structure is reached.

ELF topological analysis shows that bicyclooctatriene contains three pairs of V_1,2_(C1,C4), V_1,2_(C2,C6), and V_1,2_(C5,C8) disynaptic basins associated with the C1=C4, C2=C6, and C5=C8 electron depleted double bonds integrating to a total of 3.40 ē, 3.44 ē, and 3.40 ē, respectively, associated with the ELF-topological bond order 1.70, 1.72, and 1.70. Two disynaptic basins V(C1,C5) and V(C3,C7) are associated with the electron rich single bond C1–C5 with basin population 2.22 ē (ELF-topological bond order 1.11) and electron depleted single bond C3–C7 with basin population 1.80 ē (ELF-topological bond order 0.90), respectively. Four formal single bonds C2–C3, C3–C4, C6–C7, and C7–C8 are characterized by disynaptic basins V(C2,C3), V(C3,C4), V(C6,C7), and V(C7,C8), respectively. Briefly, basin populations analysis of cubane along the cage opening step shows that during the reaction course C1–C2, C4–C8, and C5–C6 single bonds are cleaved while the C1–C4, C2–C6, and C5–C8 bonds are saturated.

**Fig. 6:**
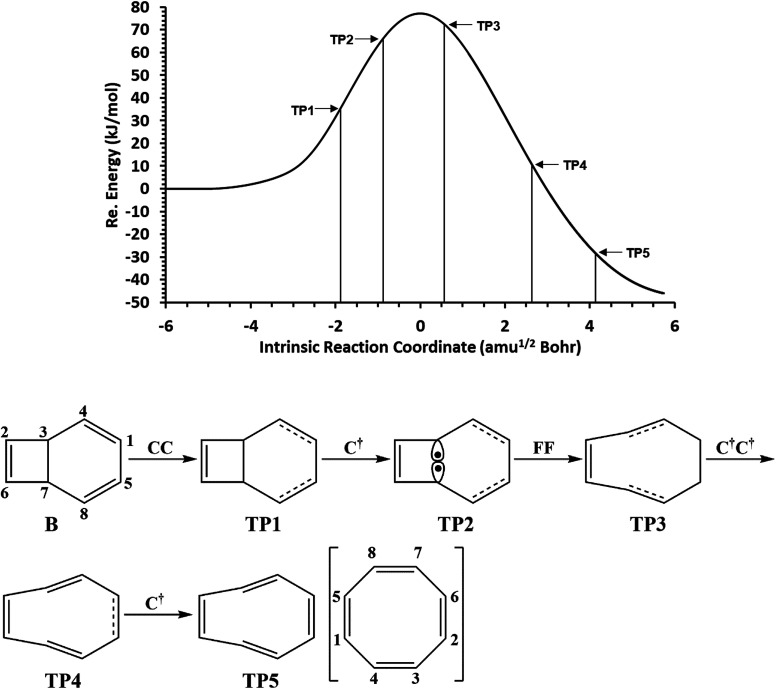
The IRC profile for the thermal rearrangement step calculated at the B3LYP/6-311G(d,p) level of theory with marked turning points and classical representation of the reaction mechanism.

**Fig. 7:**
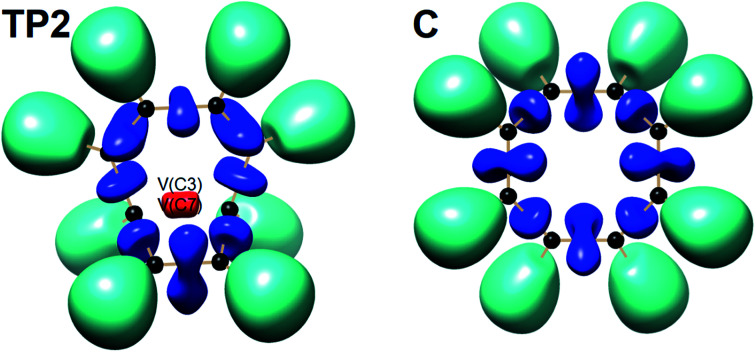
Snapshot of ELF-localization domains (*η* = 0.80) for [8]annulene and TP2 associated with the C3–C7 bond rupture in the thermal rearrangement step.

The total electronic energy profile along the thermal rearrangement of bicyclooctatriene to [8]annulene and classical representation for the chemical bonds at respective turning points are shown in Fig. 6. The snapshot of ELF localization domains for [8]annulene and turning point associated with the C3–C7 bond breaking are presented in Fig. 7. The first turning point TP1 in the thermal rearrangement step takes place at *s* ≈ −1.87433 amu^1/2^ Bohr by means of two concurrent cusp (CC) type catastrophes. The pairs of V_1,2_(C1,C4) and V_1,2_(C5,C8) disynaptic basins are transformed into two single disynaptic basins V(C1,C4) and V(C5,C8). These catastrophes reflect the topological signature change of C1=C4 and C5=C8 double bonds to the related single bonds. Meanwhile, along the first domain V(C3,C7) disynaptic basin is depopulated from 1.80 ē to 1.56 ē (see Table 5) to be ready for C3–C7 bond rupture in the next domain. The second turning point, TP2, connecting SSD II and SSD III is found at *s* ≈ −0.87977 amu^1/2^ Bohr and a cusp (C^†^) type catastrophe is observed. The C^†^ type catastrophe leads to the splitting of V(C3,C7) disynaptic basin into two monosynaptic basins V(C3) and V(C7) with populations of 0.66 ē. This is chemically equals to the C3–C7 bond breaking and formation of pseudoradical centers at the C3 and C7 atoms. When the transition structure is reached and left behind, the formed monosynaptic basins V(C3) and V(C7) are annihilated at *s* ≈ 0.57376 amu^1/2^ Bohr by means of two simultaneous fold (FF) type catastrophes. These catastrophes can be interpreted as destruction of pseudoradical centers at the C3 and C7 atoms. During this domain, disynaptic basins V(C1,C4) and V(C5,C8) are depopulated from 3.18 ē to 2.75 ē. The diminished populations are transferred to the disynaptic basins V(C3,C4) and V(C7,C8) to reach 3.10 ē and disynaptic basin V(C1,C5) to reach 2.84 ē. The enriched disynaptic basins V(C3,C4) and V(C7,C8) are replaced by two pairs of disynaptic basins V_1,2_(C3,C4) and V_1,2_(C7,C8) accounting for the C3=C4 and C7=C8 double bonds formation at *s* ≈ 2.63937 amu^1/2^ Bohr. This turning point is the endpoint of SSD IV and described by two simultaneous cusp (C^†^C^†^) type catastrophes, which is the most energetic domain that releases 61.87 kJ mol^−1^. Coincidentally, the disynaptic basins V(C1,C4) and V(C5,C8) are depopulated from 2.75 ē to 2.30 ē and the subtrahend electrons are transferred to the disynaptic basin V(C1,C5) and the newly formed pairs of disynaptic basins. Finally, at *s* ≈ 4.13121 amu^1/2^ Bohr, the turning point TP5 indicates a cusp (C^†^) type catastrophe in the region between C1 and C5 atoms.

**Table 5:** Population of the ELF-localization basins associated with the thermal rearrangement step calculated at the B3LYP/6-311G(d,p) level

	B	TP_1_	TP_2_	TP_3_	TP_4_	TP_5_	C
V_1_(C_1_,C_4_)	1.75	3.30	3.18	2.75	2.30	2.20	2.16
V_2_(C_1_,C_4_)	1.65	—	—	—	—	—	—
V_1_(C_1_,C_5_)	2.22	2.31	2.43	2.84	3.25	1.89	1.86
V_2_(C_1_,C_5_)	—	—	—	—	—	1.47	1.57
V(C_2_,C_3_)	1.95	1.97	2.00	2.06	2.10	2.12	2.16
V_1_(C_2_,C_6_)	1.74	1.74	1.74	1.76	1.77	1.81	1.86
V_2_(C_2_,C_6_)	1.70	1.72	1.73	1.73	1.72	1.65	1.57
V_1_(C_3_,C_4_)	2.04	2.14	2.28	3.10	1.81	1.84	1.86
V_2_(C_3_,C_4_)	—	—	—	—	1.55	1.57	1.57
V(C_3_,C_7_)	1.80	1.56	—	—	—	—	—
V_1_(C_5_,C_8_)	1.75	3.30	3.18	2.75	2.30	2.20	2.16
V_2_(C_5_,C_8_)	1.65	—	—	—	—	—	—
V(C_6_,C_7_)	1.95	1.97	2.00	2.06	2.10	2.12	2.16
V_1_(C_7_,C_8_)	2.04	2.14	2.28	3.10	1.81	1.84	1.86
V_2_(C_7_,C_8_)	—	—	—	—	1.55	1.57	1.57
V(C_3_)	—	—	0.66	—	—	—	—
V(C_7_)	—	—	0.66	—	—	—	—

This catastrophe leads to the creation of a pair disynaptic basins V_1,2_(C1,C5) from single disynaptic basin V(C1,C5), integrating to a total of 3.36 ē which accounts for the signature change of C1–C5 single bond to a double bond. During the last domain, the formed double bonds are populated, and subsequent electronic relaxation takes place to reach the [8]annulene. The [8]annulene can be topologically described by four pairs of disynaptic basins V_1,2_(C1,C5), V_1,2_(C2,C6), V_1,2_(C3,C4), and V_1,2_(C7,C8), integrating to a total of 3.43 ē of each (ELF-topological bond order 1.72) associated with the electron depleted double bonds C1=C5, C2=C6, C3=C4 and C7=C8, respectively. Also four electron rich single bonds C1–C4, C2–C3, C5–C8, and C6–C7 with basin population 2.16 ē of each (ELF-topological bond order 1.08) are characterized by disynaptic basins V(C1,C4), V(C2,C3), V(C5,C8), and V(C6,C7), respectively. In a word, the rearrangement of bicyclooctatriene may be explained by C3–C7 bond rupture, depopulation of C1–C4 and C5–C8 double bonds, and saturation of C1–C5, C3–C4, and C7–C8 bonds.

**Scheme 2:**
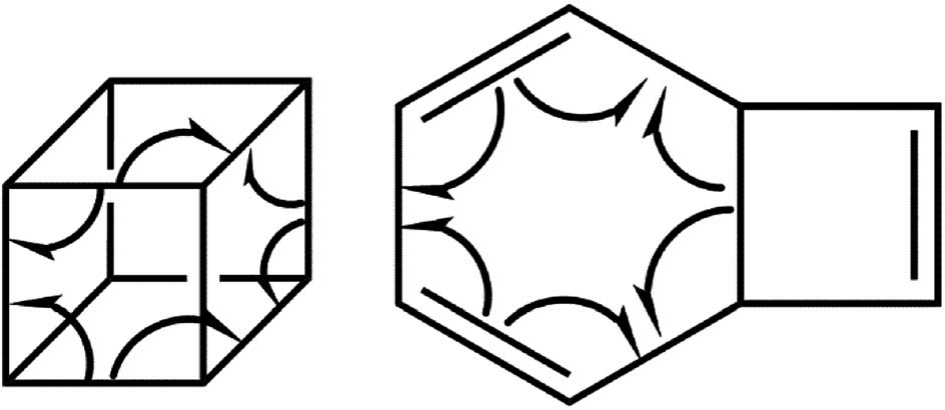


According to the ELF analysis, the molecular mechanism of the ring opening and thermal rearrangement steps can be sketched as depicted in Scheme 2, and the sequence of the turning points for the thermal pyrolysis of cubane can be represented as:*η*-1-13-C^†^C^†^FFFC^†^C^†^FFFC^†^C^†^-2-6-[C]_2_C^†^[F]_2_[C^†^]_2_C^†^-0

The curly arrows in Scheme 2 stand for bond breaking/forming and electron density rearrangement for ring opening of cubane and thermal rearrangement of bicyclooctatriene. Appearing of the turning points along reaction paths at different coordinates indicate that the electron density rearrangement along the ring opening and thermal rearrangement steps are asynchronous.

## Conclusion

The kinetic and molecular mechanism for the gas phase pyrolysis of cubane were discussed using quantum chemical methods. The pyrolysis of cubane can take place *via* different pathways. In this research, two successive pathways which lead to the formation of [8]annulene as the experimentally verified major product have been studied. A comparison between the available experimental outcomes with the results of CBS-QB3 and MN15-L/maug-cc-pVTZ level of theories indicates that the former level can accurately predict energetic results. The pyrolysis of cubane at the experimentally employed temperature of 520 K occurs *via* the cage opening of cubane to reach the bicyclooctatriene and then thermal rearrangement of bicyclooctatriene to produce the experimentally major product [8]annulene. Both pathways are exergonic and exothermic, in such a manner that the cage opening step is both enthalpy and entropy driven process while the thermal rearrangement of bicyclooctatriene is just an enthalpy driven process. The pressure dependence of the rate constants shows that the atmospheric pressure rate constant for the cage opening step lies in the high pressure limit while for the thermal rearrangement step is located at the fall-off region. Based on the kinetic results, the cage opening is the rate-determining step. The temperature dependence of the rate constant for the cage opening step at the MN15-L level can be expressed as log(*k*/s^−1^)^1bar^_MN15-L_ = (15.63) − (48.99 kcal mol^−1^)/*RT* ln 10.

The molecular mechanism of the reactions has been studied by means of the BET at the B3LYP/6-311G(d,p) level of theory. In summary, during the cage opening course C1–C2, C4–C8, and C5–C6 single bonds are cleaved while the C1–C4, C2–C6, and C5–C8 bonds are saturated. The rearrangement of bicyclooctatriene takes place *via* C3–C7 bond rupture, depopulation of C1–C4 and C5–C8 double bonds, and saturation of C1–C5, C3–C4, and C7–C8 bonds. Electron density rearrangement along the ring opening and thermal rearrangement steps are asynchronous, and the sequence of the catastrophes was represented as:*η*-1-13-C^†^C^†^FFFC^†^C^†^FFFC^†^C^†^-2-6-[C]_2_C^†^[F]_2_[C^†^]_2_C^†^-0

## Conflicts of interest

The authors declare that there is no conflict of interest.

## Supplementary Material
